# The Beliefs, Myths, and Reality Surrounding the Word Hema (Blood) from Homer to the Present

**DOI:** 10.1155/2010/857657

**Published:** 2010-07-27

**Authors:** John Meletis, Kostas Konstantopoulos

**Affiliations:** First Department of Internal Medicine, School of Medicine, Laiko General Hospital Athens, National and Kapodistrian University of Athens, Athens 11527, Greece

## Abstract

All ancient nations hinged their beliefs about hema (blood) on their religious dogmas as related to mythology or the origins of religion. The Hellenes (Greeks) especially have always known hema as the well-known red fluid of the human body. Greek scientific considerations about blood date from Homeric times. The ancient Greeks considered hema as synonymous with life. In Greek myths and historical works, one finds the first references to the uninterrupted vascular circulation of blood, the differences between venous and arterial blood, and the bone marrow as the site of blood production. The Greeks also speculated about mechanisms of blood coagulation and the use of blood transfusion to save life.

## 

**“…I*ητρική*, *δεπάνταπάλαιυπάρχει*, *κα*ί *αρχήκα*ί o*δ*ό*ςευρημένη*,* **καθ*'*ήνκα*ί *τάευρημέναπ*o*λλάτεκα*ί *καλώςέχ*o*νταεύρηταιενπ*o*λλώ*, *χρ*ό*νω*,* **κα*ί *τάλ*o*ιπάευρεθήσεται*, *ήντ*ί*ςικαν*ό*ςτεώνκα*ί *τάευρημέναειδώς** **εκτ*o*ύτων* o*ρμώμεν*o*ςζητέη*…”* *“…all these requisites belong of old to Medicine, and an origin and way have been found out,* *by which many and elegant discoveries have been made, during a length of time,* *and others will yet be found out, if a person possessed of the proper ability,* *and knowing those discoveries which have been made,* *should proceed from them to prosecute his investigations…”** *(Hippocrates, On Ancient Medicine)*

According to many linguists, the Greek word *AIMA *(*haema*, *hema,* blood) is derived from the ancient Greek verb * “*α*ί*θω*” (aetho),* which means “make red-hot, roast” “warm or heat”. In most ipsissina verba, according to this interpretation, the word stems from the passive present perfect of the verb *“*α*ί*θω*” (aetho) “*ήσμαι*” (hesmae).* From this we get * “*α*ί*σμα*” (haesma)* or *“*α*ί*μα*” (haema),* which means literally “hot” or “incandescent” (as the body fluid is supposed to be) [1]. There are some 1300 words in the English dictionary containing the Greek word “hema” and its derivatives. Many words are unedited or compound Greek words. Many words are Greek compounds with the prefixes hyper-, hypo-, auto-* (“auto”=self, same), *iso-* (“iso”=equal, uniform*), or adding the privative an-* (“*α*-”=without, not)). *There are also compound Greek and Latin words (Table 1) [1, 2].

Its definition according to Claudius Galenos of Pergamon (in Latin Galen or Galenus) (200-130 BC) [3–6] was as follows: * “*α*ί*μαεστ*ί *θερμ*ό *καιυγρ*ό*νενταιςαρτηρ*ί*αις*o*λιγώτερ*o*ν*, *εξ*o*ύτ*o *ζώ*o*ντρέφεται*”, “haema esti thermo kai hygron en tais arteriaes, exou to zoon trephetai” * (hema is a warm and fluid material which is less in the arteries, from which the animal is fed). 

The Greeks have always known hema as the well-known red fluid, which is seen as a bright red or crimson liquid gushing out of a wound in the human body or the body of a warm-blooded animal. The red cells are called erythrocytes *(*ερυθρ*ό=erythro=*red, **κύτταρα*=cytes*=cells) while in French the term used is hématies *(*αιμάτια*=haematia=haema corpuscles)*. The term “white blood cells” or leucocytes *(*λευκ*ό*ς*=leucos=white*) is not considered exact, but is used nonetheless because when they are examined fresh in the light of the microscope, they appear not as white, but as colourless cells *(*άχρωμ*o*ς*=achromos)* and so the correct term would be “colourless blood corpuscles” or “achromocytes” [1, 2].

Most ancient people or nations of the East (Phoenicians, Persians, Egyptians, and Hebrews) hinged their beliefs about hema (blood) on their religious dogmas to such a degree that they could be seen as directly related to mythology or the origins of religion.

Scientific considerations about blood begin only with the Greeks. The knowledge about blood in Homeric times could be summarized in the four following concepts which are valid even today: (a) The blood is so essential for life that it is considered to be the centre of life itself [7–9]. (b) Death is considered to be definitive and irrevocable when it is caused by lack of blood. As a result, clashes in which murder and blood * (“…*φ*ό*ν*o*ςτεκαια*ί*μα*”, “…phonos te kai haema”) *make their appearance are horrible [10–12]. (c) Besides red blood there is also dark blood in the body, not only in humans but also in animals. What we have here in fact is a distinction between arterial and venous blood. (d) From an anthropological, racial, ethnological, and social point of view, blood is regarded as a tribal, national and family bond. “*The Achaeans of different descent residing in an area extending from the north of Epirus to the southernmost island of Crete belong to one and the same nation because they have the same racial blood*”. Besides, the lineage blood connects the members of a family to each other, defined as *”ό*μαιμ*o*ν*”, “homaemon”* (with the same blood) by Herodotus (c.484-c.425 BCE). Finally, he who is courageous, valiant, and virtuous is characterised as being of *“*α*ί*ματ*o*ςαγαθ*oίo”, “haematos agathoio”* (of good and virtuous blood) [8, 9, 13].

Concerning scientific views about blood in historic Hellas, the ancient Greek savants or at least some of them, considered the blood to be the same as the soul *(*ψυχή*=psyche)*, in the sense of the nonmaterial source of life otherwise called spirit *(*πνεύμα*=pneuma).* A similar fallacy is propounded by others, who maintain that this identification of haema (blood) with psyche (soul) is also to be found in the Holy Bible. The error is easy to refute as even in Homer, the Greek Gods and the monstrous daemons are *“*ανα*ί*μωνες*=anhaemones"*, that is without blood [11, 12, 14], which does not make them dead but, on the contrary, immortal (because of the absence of blood) “…and out gushed the immortal blood of the goddess, ichor, such as flow in the veins of the serene gods,* “…*ρέεδ*' *άμβρ*o*τ*o*να*ί*μαθε*ίo, *ιχώρ*, oίo*ςπερτερέειμακάρεσσιθε*oί*σι*”, “…rhee d' amvroton haema theio, ichor, hoios per te rheei makaressi theoisi” *[11, 12, 15]. It seems that the misunderstanding of later scholars can be traced to: (a) the misinterpretation of many extracts or phrases from ancient texts such as, for example, *“*α*ί*μαφασ*ί *τινεςε*ί*ναιτηνψυχήν*”, “haema fasi tines einai ten psychen”* (the soul is said to be blood)—as Aristotle says—that in some people's opinion blood is life itself [5, 6, 15–17]. For the same reason are Galenos's words *“…*α*ί*μαε*ί*ναιτηνψυχήν*”, “…haema einai ten psychen” *(*…*blood is the soul) [3, 4, 18]. (b) Finally, the most glaring of these errors is the misinterpretation of the theory of Empedocles (c.492-432 BC) “the soul, or the blood circulating around the heart, * (“*α*ί*μαγαρανθρώπ*o*ιςπερικάρδι*o*νεστ*ί *ν*ό*ημα*”, “haema gar anthropois pericardion esti noema”)* as it identified the soul with the blood [19, 20]. All the above would be true if we understood *“psyche”* as synonymous with “life” and more specifically “earthly life,” which explains why some identified blood with the material cause of physical life, in contrast with the majority of savants who considered blood to be the centre of physical life.

According to the most widespread ancient Greek scientific views, the blood was believed to be a necessary nutrient of the living body, as are juices for plants. Not all animals share the same type of blood and Aristotle (384-322 BC) notes that *“…all animals are endowed with a fluid whose lack, either natural or symptomatic, causes their death. In some animals this liquid is their blood, while in others it is a colourless liquid which replaces blood” (“*έναιμ*a”, “enhaema”, *and **“**άναιμα*”, “anhaema” *i.e., with and without blood). He considers blood to be the essence of nutrition and the preservation of the body heat and calls it the ultimate food **(*“*έσχατητρ*o*φή*”, “eschate trofe”)*, that is food in the most perfect form which creates and preserves the noblest parts of the body (the nature of man consists of the most pure substances that is, the blood, the flesh, and the other sensory organs, “**ηφύσιςεκμεντηςκαθαρώτατηςύλης* (hema) *σάρκαςκαιτωνάλλωναισθητηρ*ί*ωνσυν*ί*στησιν*…”, physis ek men tes katharotates hyles sarcas kai ton allon haestheterion synistesin…*” [15–17].

For the ancient world, the function of the heart and vessels was a great mystery. Many hundred years BC the Greeks Herophilus of Chalcedon (c.335-c.260 BC) and Alcamaeon of Croton (c.535-unknown BC) believed that arteries and veins are dissimilar in animals; arteries are thicker than veins and carry blood and made the first description of basic aspects of circulation [21, 22]. They suggested that sleep was caused by blood draining from the brain via the veins, and that death was the result of the brain becoming completely drained. Two hundred years later, Aristotle ascribed the power of thought to the heart, which he contended also contained the soul. Erasistratus (304-250 BC) understood that the heart served as a pump, thereby dilating the arteries, and he found and explained the role and function of the heart valves. He proposed that the arteries and veins both spread from the heart, dividing finally into extremely fine capillaries that were invisible to the eye. However, he believed that the liver formed blood and carried it to the right side of the heart, which pumped it into the lungs and from there to the rest of the body's organs. He also believed that pneuma, vital spirit, was drawn in through the lungs to the left side of the heart, which then pumped the pneuma through the arteries to the rest of the body. Galenos demonstrated the inaccuracies of many of these theories, demonstrating that the arteries carried blood rather than air, using animal dissections. Thus he proposed the theory that blood flowed back and forth within the arteries [23]. 

Plato (427-347 BC) in Timaeus mentioned that the heart is the source of the blood which vigorously circulates through all parts of the body * “…*κατάπάνταταμέλητ*o*υσώματ*o*ςσφ*o*δρώςπεριφερ*o*μέν*o*υα*ί*ματ*o*ς*…”, *“*…kata panta ta mele tou somatos sphodros peripheromenou haematos…”* [24], while Aristotle had already concluded that the blood must be uninterruptedly circulating within the blood vessels, periodically returning to its starting point the heart [15–17]. Long before Galenos, Hippocrates (c450-c.380 BC) already knew that the blood moves and performs a periodic or circular movement * “*εκτελε*ί *περ*ίo*δ*o*νήκύκλ*o*ν*”, “periodon, cyclon” *[5, 6, 25, 26]. Hippocrates also believed that blood is produced in the liver and spleen and travels to the heart and gets warmed or cooled in the lungs, in addition to the knowledge that the human body has four types of fluid (blood, phlegm, black, and yellow bile) [27]. Galenos accurately describes the small blood circulation and suspects the previous while differentiating between venous and arterial blood. He used for the first time the word hematopoietic and also made the differentiation of venous blood (dark blood) originating from liver from the arterial blood (red blood) originating from the heart. He believed also that the blood is produced in the liver, travels through veins to body parts, and passes between ventricles through pores in the interventricular septum [21, 22, 28]. He also suspected the function of the breath during which the blood receives from the inhaled air not the air itself* “…o*υχ*ί*των* o*υσιώναυτ*o*ύ*”, “…ouchi ton ousion autou*” **but a familiar and friendly quality therein * “…o*ικε*ί*ανκαιφ*ί*ληνπ*o*ι*ό*τητα*”, “…oikeian kai philen poioteta”* [3, 4, 29] which is a prerequisite for life, the breath of life **“**ζωτικ*ό*νπνεύμα*”, “zotikon pneuma” (spiritus vitalis or life spirit)* as he called it, originating in the heart and flowing through the arteries [3, 4, 30]. This is what Lavoisier later called oxygen. However, Galenos also believed that life was sustained by food, which turned into blood in the liver, which nourished the heart, lungs, the brain, and other organs. Waste materials were also thought to be removed by the blood. Thus, the blood circulation and metabolism are significant elements of his philosophical theory and he was a pioneer in suggesting a relationship between food, blood, and air [3, 4].

The contribution of other Nations and scientists in the field of circulation, blood components, coagulation, and blood transfusion was very significant in the next centuries as well. The Syrian Ibn Nafis (1210-1288 AD) first described the pulmonary circulation [22, 31], the Iranian Rhazes (865-925 AD) made an accurate description of the heart valves [28, 32], the British William Harvey (1627) showed that the blood circulated within a closed system and described the mechanisms of both systemic and pulmonary circulation in humans [28], and the Italian Marcello Malpighi (1658) made the first description of capillary circulation [28]. The first accurate descriptions of red blood cells were made by Dutch Jan Swammerdam (1630) and Antony van Leeuwenhoek (1674). The German Ernest Christian Neuman (1866) demonstrated that erythropoiesis and leukopoiesis formulate in the bone marrow and described the presence of nucleated red blood cells in bone marrow and proposed, in opposition to P. Ehrlich, in view of his one-stem-cell-theory for all blood cell lines even in extrauterine life. In this period, nobody noticed this idea (beside Pappenheim), which became such an importat fact today. On the basis of his observation, Ernst Neumann was the first to postulate the bone marrow as blood forming organ with a common stem cell for all hematopoietic cells [33]. The British William Osler (1870) made the first description of platelets, formation of blood clots, and hints of possible synthesis in bone marrow. The English William Hewson (1869) has been referred as the “father of hematology” noted that the red cells were flat rather globular and also described the leukocytes for the first time and demonstrated that red blood cells were discoid rather than spherical as had been previously supposed by Anton van Leeuwenhoek, but incorrectly identified its dark center as its nucleus. In 1773 he produced evidence for the concept of a cell membrane in red blood cells, however, this last work was largely ignored [34]. The German Paul Ehrlich (1877) identified neutrophils, basophils, and eosinophils on the basis of staining of their granules. The first fully-documented blood transfusion to a human was administered by the French Jean-Baptiste Denis (1667) when he transfused the blood of a sheep into a 15-year-old boy and the first human to human transfusion by British James Blundell (1818). The Austrian Karl Landsteiner (1901) documented the first three human blood groups (A, B, and O) and the American Reuben Ottenberg (1907) performed the first blood transfusion using blood typing and cross-matching [21–23, 28, 31, 32].

In the last century, the progresses are very impressive concerning the clinical as well as the experimental fields of hematology. The Americans Thomas Benton Cooley, Pearle Lee, and coworkers (1925) described thalassemia as a particular entity, the Austrian-English Maxwell Myer Wintrobe (1929) describes the method of obtain the hematocrit by centrifusion of blood in a glass tube and defines MCV, MCH, and MCHC, and the American Wallace H. Coulter developed the electronic instrument for measuring the blood cell parameters of all blood cells. In the Mid-1960s the Canadians James Till and Ernest McCulloch (1961), the Australians Ray Bradley and Don Metcalf (1966), and the English Mike Dexter and coworkers (1977) developed the cultures of hematopoietic progenitor cells and the Canadians Bill Robinson and Beverely Pike and coworkers (1970s) made progresses for the recognition and use of colony stimulating factors in the stem cell research. At the end of 1960's the teams of the American E. Donnall Thomas and the French George Maté made the first attempts of bone marrow transplantation especially for leukemia after failure of all other treatments. The bone marrow transplantation technique was significantly assisted after HLA discovery by J. Dausset early in 1970's and at the ends of 1980s the team of French Eliane Gluckman made the first successful umbilical cord blood transplantation as an alternative source of stem cells. The research results of the last year have been ameliorated the pathophysiology, diagnosis, and management of all hematological disorders benign and malignant [35–41].

Blood plays a significant role in Greek mythology, too, although the Greek Gods did not normally like blood sacrifices, which differentiates them from other ancient cultures. In mythology, the blood seems to have been something related to the psyche and the spirit, as one can easily conclude from primeval beliefs among savages and semisavages, who sprinkled the entrances to their caves or huts with it. This action is due to the belief that demons not only feed on blood but have an insatiable thirst for it and are attracted by its smell (hence their satisfaction by the blood sprinkled at entrances) [42–44]. This belief was handed down to historical times and determined the role of blood in blood sacrifices.

Already in Homer's Iliad it is first mentioned that the type of blood is passed on to the descendants, * “…*ανδρώνγενεής* oί *δ*' *α*ί*ματ*o*ςεξεμ*o*ύεισ*ί”, “…andron geneis oi d' haematos ex emou eisi” *(*…*men of my lineage who have the same blood as mine) [10–12, 45] and * “…*ανδρών* oί*τηςεξα*ί*ματ*o*ςεισ*ί*γενέθλης*”, “…andron oitis ex haematos eisi genethlis” *(*…*men of your own blood lineage) [10–12, 46], while the distinction between venous and arterial blood is also clearly drawn, * “…*ρέεδ*' *α*ί*μακελαινεφές*…”, “…ree d' haema kelainephes…” *(*…*dark blood was flowing) [7–9, 47], *“…*σύτ*o*δ*' *α*ί*μακελαινεφές*…”, “…syto d' haema keleinephes…”* (*…*dark blood was gushing out) [10–12, 48], and * “…*εκθ*' *α*ί*μαμέλανρέει*…”, “…ek th' haema reei” *(*…*from the wound black blood was flowing) [10–12, 49]. In the Iliad we also read for first time about the coagulation of blood after being shed, **“*…*α*ί*μαπαχύπτύ*o*ντα*…”, “…haema pachy ptyonta…” *(*…*shedding thick blood*…*) [10–12, 50] and * “…*βρ*ό*τ*o*ναιματ*ό*εντα*”, “…broton haematoenta” *(*…*congealed blood) [10–12, 51]. Besides, the Odyssey mentions that blood is produced in the bone marrow which is also considered to be the regulator of human affect, *“…*τηςδ*' *επε*ί*εκμέλανρ*ό*η*, *λ*ί*πεδ*'o*στέαθυμ*ό*ς*”, “…tes d' epei ek melan roe, lipe d' ostea thymos”* (*…*as soon as black blood started flowing from the wound, vigour left the bones) [7–9, 52]. There is one point in Homeric mythology which is uncannily similar to modern hematology and more specifically with blood transfusions, namely, that the only one way for the “shadows” of the dead to recover their senses and be brought back to life is by being enriched with blood, even sheep's blood [8, 9, 53]. In this extract, Odysseus uses two sheep, sorceress Circe's gift, to bring the soothsayer Teiresias back to life, so that he can ask him about his companions and about the day of his return. Blood transfusions also seem to originate in the therapeutics of Galenos, who first raised the question of finding ways of “getting rid of the corrupt blood in a human body and infusing healthy blood in its place”** (*“*ενανθρώπ*o*υσώματικενώσαιμεντ*o *διεφθαρμέν*o*να*ί*μα*, *χρηστ*ό*νδεεπεγχέειν*”, “en anthropou somatic kenosae men to diephtharmenon haema, christon de epencheein”)* [3, 4, 54]. These views, however, happen to be shared by last century poetry. Thus, by bringing back to life, blood reactivates memory, being its prerequisite. In this context, a famous Greek poet, Lorentzos Mavilis, (1860–1912), wanting to safeguard peace and quiet for the dead, presents them as drinking “the water of oblivion”* “*ύδωρλησμ*o*νιάς*”, “hydor lesmonias”* from a crystal fountain, and not blood *(“*τέτ*o*ιανώραν* o*ιψυχέςδιψ*o*ύνκαιπάνεστηςλησμ*o*νιάςτηνκρ*o*υσταλλένιαβρύση*”) *[55].

Evidently, this connection between blood and the metaphysical dimension in human reality is evidently not Homer's monopoly. One finds it in later texts as well. Hippocrates, for example, relates the biological functions of blood to human idiosyncrasy and mentions the four humours or bodily fluids [blood, phlegm, choler (yellow), and black bile * “*μέλανχ*o*λή*”, “melan chole” *(melancholy)]—blood mutations which he considers responsible for man's health, sickness, or even behavioural patterns. According to Galenic medicine, in the case of “humor sanguinicus” (presumably an undesirably cheerful and lively temperament resulting from the dominance of the blood), doctors needed to bleed them to restore their patients' physiological balance. He also relates the humors to the seasons of the year [3, 4, 25, 26]. This treatise is also the first attempt to categorize human character. Later on, in mythological texts which, having originated in Greece and spread eastwards with Alexander, were brought back to their birthplace by the Arabs and the Byzantines, one sees the same views about blood. The latter views bear such a striking resemblance to the former that the two cannot possibly be unrelated, thus opening the way to both medical and comparative literature historical research. 

It is enjoyable to read about a Persian Zoroastrian doctor Perzhoe (Borzhoa in Persian) who was sent to India in the 6th century AD to learn the secrets of their philosophers and bring them to his king, Chosroes I, in order for the king to become wiser in his governance. The most important thing Perzhoe learns while copying and translating these texts is to be in constant discourse with his soul—the result of a smooth coordination between “his blood and his bile” [56]. This harmony between body and soul, the *krasis* (Greek for “mixture,” later “good health”), influences him to such a degree that he turns to introspection and philosophy, as he considers this to be the only way in which he can be a competent healer. Also during this internal discourse, he kept saying: “*Oh soul, avoid the ephemeral, pursue the eternal and be self-sufficient; and be as virtuous as you can; and remember the constitution of your body, that it can be taken ill, that it can die, that the clay vessel of the body is filled with four fluids in delicate balance with each other and while these fluids coexist, it has life; if one of them is less or more than needed, the body collapses and dies. And just as a wooden effigy collapses when you take away the nails that hold its parts together, so does the human body if you disturb its krasis” *[56]. 

This extract alone would suffice to show the significance attached by savants (since Hippocrates's times to this day, as is evident in many later authored or folk texts, of the Byzantine period in particular) to the *krasis* of the blood and the other fluids in the function and constitution of the body and to the influence this awareness could have on a doctor of the period who would be keen on achieving true maturity and wisdom. Even today the boundaries between the physician and the philosopher of medicine remain blurred.

## Figures and Tables

**Table 1:** Examples of words in the english dictionary containing the greek word “hema” and its derivatives.

(1) Unedited or compound Greek words
(i) Hematemesis* (H. +G. “emesis”=vomiting), *
(ii) Hematocrit *(“hema” +G. “krites”=judge), *
(iii) Hemapheresis *(H. +G. “apheresis”=removing), *
(iv) Hemodialysis *(H. +G. “dialysis”=dissolution), *
(v) Hemochromatosis *(H. +G. “chroma”=color, “chrosis”=coloration), *
(vi) Hemolysis *(H. +G. “lysis”=disintegration of cells)*,
(vii) Hemopoiesis *(H. +G. “poio”=to produce), *
(viii) Hemorrage *(H. +G. “rhegnymae”=to burst forth), *h
(ix) Hemostasis *(H. +G. “stasis”=halt), *
(x) Oligemia *(G. “oligos”=few, little + H.), and so forth. *

(2) Greek words compounds with
(a) The prefixes hyper- *(G. “hyper”=over, above, excessive, beyond) *
(i) Hyperglycemia *(G. “hyper” +G. “glykys”=sweet +G. “hema”) *
(ii) Hyperuricemia* (G. “hyper” +G “ourico”=uric +G. “hema) *
(b) The prefixes hypo- *(“G. “hypo”=below, beneath, under, less than normal) *
(i) Hypoxemia *(G. “hypo” +G. “oxy”=oxygen +G. “hema” ) *
(ii) Hypokalemia *(G. “hypo” +G. “kalio”=pottasium +G. “hema”) *
(c)* The prefixes *auto-* (G. “auto”=self, same), *
(i)* Autohemolysis (G. “auto” + H. +G. “lysis”=gradual decline) *
(d)* The prefixes *iso-* (G. “iso”=equal, uniform*)
(i)* Isohemolysis (G. “iso” + G. H. +G. “lysis”=gradual decline)) *
(e) Adding one privative a-* (G. “*α*-”=without, not) *
(i) Anemia* (“a-” + G. “hema”). *
(f) Compound Greek and Latin words
(i) Hemoglobin (*“hema”. +L. “globin”= G. “spherine”=globus, globulin), *
(ii) Immunohemolysis *(G. “anoso”= L. “immuno” + G. “hema” +G. “lysis”=gradual decline), and so forth. *

G. Greek word,

H. Hema,

L. Latin word
